# Dual Guided Aggregation Network for Stereo Image Matching

**DOI:** 10.3390/s22166111

**Published:** 2022-08-16

**Authors:** Ruei-Ping Wang, Chao-Hung Lin

**Affiliations:** Department of Geomatics, National Cheng-Kung University, Tainan City 701, Taiwan

**Keywords:** dense image matching, deep learning, left–right consistency

## Abstract

Stereo image dense matching, which plays a key role in 3D reconstruction, remains a challenging task in photogrammetry and computer vision. In addition to block-based matching, recent studies based on artificial neural networks have achieved great progress in stereo matching by using deep convolutional networks. This study proposes a novel network called a dual guided aggregation network (Dual-GANet), which utilizes both left-to-right and right-to-left image matchings in network design and training to reduce the possibility of pixel mismatch. Flipped training with a cost volume consistentization is introduced to realize the learning of invisible-to-visible pixel matching and left–right consistency matching. In addition, suppressed multi-regression is proposed, which suppresses unrelated information before regression and selects multiple peaks from a disparity probability distribution. The proposed dual network with the left–right consistent matching scheme can be applied to most stereo matching models. To estimate the performance, GANet, which is designed based on semi-global matching, was selected as the backbone with extensions and modifications on guided aggregation, disparity regression, and loss function. Experimental results on the SceneFlow and KITTI2015 datasets demonstrate the superiority of the Dual-GANet compared to related models in terms of average end-point-error (EPE) and pixel error rate (ER). The Dual-GANet with an average EPE performance = 0.418 and ER (>1 pixel) = 5.81% for SceneFlow and average EPE = 0.589 and ER (>3 pixels) = 1.76% for KITTI2005 is better than the backbone model with the average EPE performance of = 0.440 and ER (>1 pixel) = 6.56% for SceneFlow and average EPE = 0.790 and ER (>3 pixels) = 2.32% for KITTI2005.

## 1. Introduction

Estimating depths from stereo images is a fundamental and important research topic in photogrammetry and computer vision with many applications, including 3D model/scene reconstruction, autonomous driving, robotics, and topographic mapping. The task of stereo matching is searching for the correspondences between pixels in stereo images and calculating their disparities. According to the workflow in [1], a typical stereo matching process consists of four main steps, namely the matching cost computation, cost aggregation, disparity optimization, and disparity refinement. Each step in this pipeline has been extensively studied, and several advanced methods have been proposed [2,3,4,5,6]. Although this workflow performs well, the stepwise pipeline lacks an overall objective function for global optimization, and thus may suffer errors in each step [7].

Recently, deep learning techniques have shown remarkable performance in various tasks. In stereo matching, convolutional neural networks (CNNs) were first utilized by Žbontar and Lecun [8] in matching cost computation. Instead of using similarity metrics, the authors proposed a Siamese CNN to measure the similarity between image patches. Several deep-learning-based methods have also addressed the problem of generating unary terms as similarity measurements using CNN [9,10,11]. These methods require post-processing to produce disparities. Therefore, Mayer et al. [12] proposed the integration of all steps into an end-to-end network and directly estimated the disparity from stereo images. Recently, Zhang et al. [13] proposed a guided aggregation network (GANet). Inspired by the cost aggregation in [2], a semi-global aggregation layer, which is a differentiable approximation of the semi-global matching, was introduced to capture the cost dependencies of the entire images. Xia et al. [14] showed that GANet outputforms semi-global matching by a significant margin, even when the training and validation data originate from different domains.

Although the deep-learning models for stereo matching have an excellent performance, current models still face the challenges of dealing with object occlusions. Taking advantage of disparity information from the left-to-right and right-to-left mutual disparity curves is an effective strategy to reduce the number of mismatched pixels. Applying a learning-based method to predict the correctness of output disparities [15,16] is another way to remove mismatched pixels. However, the traditional left–right consistency check and the learning-based disparity correctness prediction address the removal of mismatched pixels rather than the improvement of disparity estimation accuracy. Similarly to the idea in the studies [17,18,19] that estimate disparity confidences and inject the estimated confidences into the disparity optimization stage, we integrated left-to-right and right-to-left image matchings in the model training and utilize the mutual disparity curves from dual cost volumes to reduce the possibility of pixel mismatch. In this scenario, the left–right consistency check or the learning-based confidence estimation is regarded as an optional step in post-processing for highly confident disparity extraction. The GANet is selected as the backbone, and the proposed dual scheme can be applied to other advanced stereo matching models. To integrate the left-to-right and right-to-left image matchings in a network, a cost volume consistentization with flipped training is proposed to unify the initial cost volumes for both matchings and to learn invisible-to-visible matchings in addition to general visible-to-invisible matching. Moreover, a suppressed multi-regression is attached with the Dual-GANet to remove unrelated information in regression and to provide support for multiple disparity candidates. Compared with related methods, the main contribution of the current study is to propose a dual guided aggregation network for stereo image matching. Through a consistentization process on initial cost volumes, a Siamese CNN attached with suppressed multi-regression is established, which can reduce the mismatching possibility and support multiple candidate selection. The remainder of the paper is organized as follows. The related work is described in Section 2, and the methodology is presented in Section 3. The experimental results and conclusions are provided in Section 4 and Section 5, respectively.

## 2. Related Work

Many stereo image matching methods have been proposed in the literature, and only the deep-learning-based methods related to this study are reviewed. The related deep-learning-based stereo matching methods are classified into three categories: matching cost learning, end-to-end disparity learning, and left–right consistent learning. The reviews of related methods in these three categories are described in the following subsections.

### 2.1. Matching Cost Learning

In this category, matching learning is utilized to compute the matching cost of image patches. In [8,11], a Siamese network containing stacked convolutional layers is used to learn a similarity measurement of image patches. Similarly, Luo et al. [10] proposed a Siamese network to learn a probability distribution over all disparity values, after which they used a dot-product layer to join the branches of the network. Chen and Yuan [9] proposed a multi-scale CNN, in which the global context in down-sampled image patches is utilized to improve feature description. The methods based on matching cost learning follows the typical workflow introduced in [1], which makes the methods applicable for datasets in previously unseen domains. However, the stepwise workflow lacks an overall objective function for global optimization.

### 2.2. End-to-End Disparity Learning

The idea is integrating all steps in the typical stereo matching workflow into a neural network and training the network in an end-to-end manner. Mayer et al. [12] created a large synthetic dataset called Flying Chairs to train end-to-end deep networks for disparity estimation. Dosovitskiy et al. [20] proposed an encoder-decoder network called FlowNet to estimate disparity. Based on FlowNet, several studies have improved its performance by stacking multiple networks [21]. Kendall et al. [22] proposed a new network called GCNet, which utilizes 3D convolutional filters to regularize the cost volumes and adopts a regression loss instead of a classification loss. Similarly to GCNet, the PSMNet proposed by Shaked and Wolf [23] uses spatial pyramid pooling and 3D convolution to incorporate contextual information in different scales. Recently, Zhang et al. (2019) [13] proposed GANet, which is inspired by the cost aggregation in [2]. A semi-global aggregation layer is presented to capture the cost dependencies of images. The CSPN proposed by Cheng et al. [24] is a linear propagation model. The propagation is performed using recurrent convolutional operations, in which the affinity among neighboring pixels is learned through a deep CNN. Considering the computation cost and memory limitation, Yang et al. [25] proposed an end-to-end and coarse-to-fine hierarchy framework for high-resolution stereo images. Based on those end-to-end disparity learning networks, in this study, a left–right consistent matching scheme is proposed, which utilizes a consistentization process to generate mutual disparity curves for mismatch reduction.

### 2.3. Left–Right Consistent Learning

Inspired by the left–right consistency check, Jie et al. [26] proposed a left–right comparative recurrent model based on a convolutional long-short term memory (LSTM) network. The model contains two parallel stacked convolutional LSTM networks for left-to-right and right-to-left matchings, respectively. At each recurrent step, the model processes both views in parallel and produces error maps by performing left–right disparity comparison. The pixel mismatch generally happens in occlusion regions, because the matchings from invisible to visible pixels are missing during model training. Therefore, in the current study, a Siamese network that is unlike the LSTM-based structure in Jie et al. [26] is adopted to learn not only the matching between two visible pixels but the matching from invisible pixels to visible pixels.

## 3. Methodology

**Figure 1:**
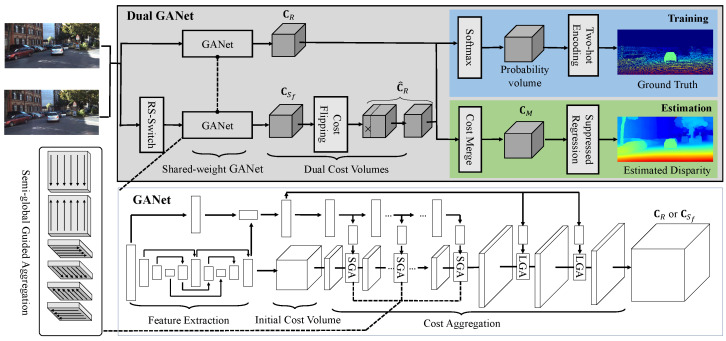
Overview of the Dual-GANet architecture. The Dual-GANet is a Siamese network containing the components of Siamese GANet, cost volume consistentization, flipped training (marked in blue), and suppressed regression (marked in green). The consistentization process consists of RS-Switch and cost flipping in the second GANet.

Figure 1 illustrates the architecture of the Dual-GANet, which is a Siamese network containing the components of cost volume consistentization, flipped training, and suppressed regression. In the network, the GANet is selected as the backbone with extensions and modifications on the semi-global guided aggregation layer, disparity regression, and loss function. A volume-guided diffusion with six directions is utilized in the cost volume determination for the enlargement of receptive fields. To generate consistent cost volumes and learn invisible-to-visible matching, a cost volume consistentization process is performed, which consists of the RS-Switch to exchange reference and support images and cost volume flipping to unify pixel positions in cost volumes. In suppressed regression, similarly to the data suppression in [27], unrelated information is suppressed before the disparity regression, and multiple disparity candidates are allowed. In this section, the extensions of the GANet, cost volume consistentization, suppressed regression, and loss function are described in Section 3.1, Section 3.2, Section 3.3, Section 3.4, respectively.

### 3.1. GANet

The GANet proposed by Zhang et al. [13] contains the components of initial cost volume construction, cost aggregation, and guidance network, all of which are inspired by semi-global matching (SGM) [2]. Unlike the traditional patch similarity measurement, the initial cost volume is constructed by iteratively concatenating features of the left and right images with an incremental disparity shift. The features are generated by a shared-weight and stacked autoencoder network. The initial cost volume is a 4D tensor of size $H\times W\times D_{max}\times 2F$, where *H* and *W* represent the height and width of the images, respectively; $D_{max}$ denotes the max disparity; and *F* represents the number of features extracted by the autoencoder network. The initial cost volume is further refined by cost aggregation, which contains semi-global guided aggregation (SGA) layers, local guided aggregation (LGA) layers, and guidance subnets.

The SGA layer is defined based on the cost aggregation in SGM. In SGM, the aggregated cost $L_{r}\left(p,d\right)$ of a pixel p with the disparity *d* in the direction r is recursively defined as

(1) $$L_{r}\left(p,d\right)=C\left(p,d\right)+\min\left\{\begin{array}{c} L_{r}\left(p-r,d\right),\\ L_{r}\left(p-r,d-1\right)+P_{1},\\ L_{r}\left(p-r,d+1\right)+P_{1},\\ \min_{i}L_{r}\left(p-r,i\right)+P_{2}, \end{array}\right.$$

where C(p,d) presents the cost of the pixel p with the disparity *d* in the initial cost volume, and $P_{1}$ and $P_{2}$ are the cost penalties to prevent discontinuities and enforce smoothness in the disparity field. The discontinuity preservation is performed by adding $P_{1}$ or $P_{2}$ to the costs to penalize large changes in neighboring disparities. In GANet, the penalty parameters are defined as learnable weights in the network. Thus, the penalties are adaptive and changeable based on neighboring context information. Following the definition in SGM, the aggregated cost in the SGA layer is formulated as

(2) $$\begin{array}{c} L_{r}\left(p,d\right)=\mathrm{sum}\left\{\begin{array}{c} w_{0}\left(p,r\right)\times C\left(p,d\right),\\ w_{1}\left(p,r\right)\times L_{r}\left(p-r,d\right),\\ w_{2}\left(p,r\right)\times L_{r}\left(p-r,d-1\right),\\ w_{3}\left(p,r\right)\times L_{r}\left(p-r,d+1\right),\\ w_{4}\left(p,r\right)\times \max_{i}L_{r}\left(p-r,i\right). \end{array}\right.\\ s.t.\sum_{i=0}^{4}w_{i}\left(p,r\right)=1, \end{array}$$

where $\left\{w_{0}\left(p,r\right),\ldots ,w_{4}\left(p,r\right)\right\}$ are the penalty weights in the network. The cost volume C(p,d) in Equation (2) can be $C_{R}\left(p,d\right)$ in the left-to-right matching or $C_{S_{f}}\left(p,d\right)$ in the right-to-left matching. The external and internal minimal cost selections in Equation (1) are replaced by weighted sums, which are implemented by using convolutions with strides, thus leading to an all convolutional network. Similarly to SGM, the values of aggregated costs $L_{r}$ increase along the path, which may lead to extremely large values. Thus, the weights $\left\{w_{0}\left(p,r\right),\ldots ,w_{4}\left(p,r\right)\right\}$ are normalized to avoid such a problem. In the implementation, the initial cost volume is sliced into $D_{max}$ slices for each disparity, and each slice performs the cost aggregation in Equation (2) with shared weight matrices.

The utilization of downsampling and upsampling in a stacked autoencoder may blur the thin structures in images. To alleviate this problem, the LGA layer containing several guided filters was used to refine the matching costs and to recover thin structures. The local aggregation is formulated as

(3) $$\begin{array}{c} L\left(p,d\right)=\mathrm{sum}\left\{\begin{array}{c} \sum_{q\in N_{p}}w_{0}\left(p,q\right)\times C\left(p,d\right),\\ \sum_{q\in N_{p}}w_{1}\left(p,q\right)\times C\left(p,d-1\right),\\ \sum_{q\in N_{p}}w_{2}\left(p,q\right)\times C\left(p,d+1\right), \end{array}\right.\\ s.t.\sum_{q\in N_{p}}w_{0}\left(p,q\right)+w_{1}\left(p,q\right)+w_{2}\left(p,q\right)=1, \end{array}$$

where q is a pixel in the K×K neighbor region of the pixel p. The LGA layer has three K×K filters with corresponding weights $w_{0}\left(p,q\right)$, $w_{1}\left(p,q\right)$, $w_{2}\left(p,q\right)$ at each pixel p for the disparities *d*, d−1 and d+1, respectively.

**Figure 2:**
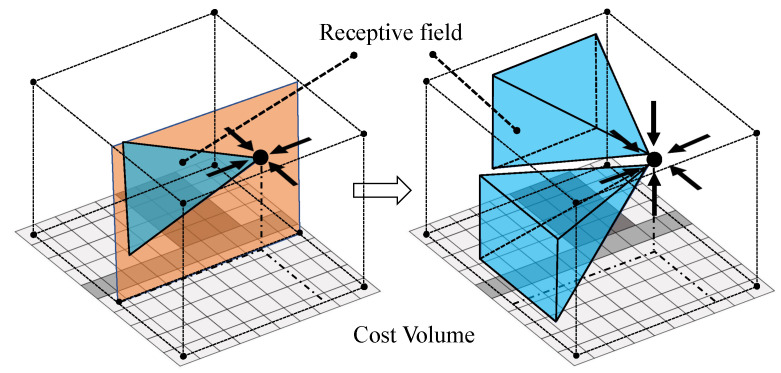
Illustration of the receptive field expansion in SGA layers. **Left**: Four-direction aggregation with 2D receptive field in the GANet. **Right**: Six-direction aggregation with 3D receptive field in Dual-GANet. The receptive fields are visualized with blue color.

Inspired by the concepts of data diffusion and the receptive field for context learning in [24,28], two modifications on the SGA layer are made in the current study. First, to increase the receptive field and to incorporate more context information in matching cost estimation, the 1D convolution for the slice aggregation in the SGA layer is extended to 2D convolution, as illustrated in Figure 2. Then, the aggregation is performed on the 3D cost volume instead of a 2D slide of the cost volume. As a result, the number of aggregation directions is increased from four to six, including left, right, forward, backward, up and down, thus leading to receptive field enlargement. Second, the internal maximal selection in Equation (2), which requires the traversal in the entry disparity axis, is removed in order to consider the computational cost. Considering these two modifications, the cost aggregation is reformulated as

(4) $$\begin{array}{c} L_{r}\left(v\right)=\mathrm{sum}\left\{\begin{array}{c} w_{0}\left(p,r\right)\times C\left(v\right),\\ \sum_{q\in N_{p}}w_{q}\left(p,r\right)\times L_{r}\left(v-r+q\right), \end{array}\right.\\ s.t.\sum_{i=0}^{k^{2}}w_{i}\left(p,r\right)=1, \end{array}$$

where $L_{r}\left(v\right)$ and $w_{q}\left(p,r\right)$ represent the aggregated cost of the voxel v and the weights for the neighbor pixel q in the direction r, respectively. Similarly, the weights are normalized to prevent numerical overflow.

### 3.2. Cost Volume Consistentization and Flipped Training

The purpose of stereo matching is to find conjugated points in a reference image based on its support image. The extracted conjugated pixel pairs can be represented as a matching set $\left\{\left(V_{R}\to V_{S}\right),\left(V_{R}\to I_{S}\right)\right\}$, where $V_{R}$ denotes the visible pixel set in the reference image, and $V_{S}$ and $I_{S}$ represent the visible and invisible pixel sets, respectively, in the support image. The extraction of the matching pairs $\left(V_{R}\to V_{S}\right)$ using matching metrics is effective because the pixels and their local neighbors are visible. However, mismatch may happen in the determination of the matching pairs $\left(V_{R}\to I_{S}\right)$ because of the occlusion and invisibility in the support image. The matching pairs $\left(V_{R}\to V_{S}\right)$ and $\left(V_{R}\to I_{S}\right)$ can be learned by a neural network with the aid of labeled training images, and the mismatches incurred by occlusion can be alleviated. However, the labeled training set did not contain the matching $\left(I_{R}\to V_{S}\right)$, that is, the matching of an invisible pixel in the reference and a visible pixel in the support image. In addition, the inference of the matching $\left(I_{R}\to V_{S}\right)$ from $\left(V_{R}\to V_{S}\right)$ and $\left(V_{R}\to I_{S}\right)$ is inefficient. To solve this problem, flipped training with Dual-GANet is proposed which contains left-to-right and right-to-left image matching. The left-to-right matching pairs provide the matching information $\left(V_{R}\to V_{S}\right)$ and $\left(V_{R}\to I_{S}\right)$, whereas the right-to-left image matching pairs support the learning of $\left(I_{R}\to V_{S}\right)$. In the implementation, the right-to-left image matching is realized by flipping the left-to-right matching. In other words, the matching examples $\left(I_{R}\to V_{S}\right)$ are created by flipping the matching $\left(V_{R}\to I_{S}\right)$ in the left-to-right matching to $\left(I_{S}\to V_{R}\right)$ in the right-to-left matching. Furthermore, a consistentization process is performed to ensure the consistency of the cost volumes generated from the left-to-right and right-to-left matching.

**Figure 3:**
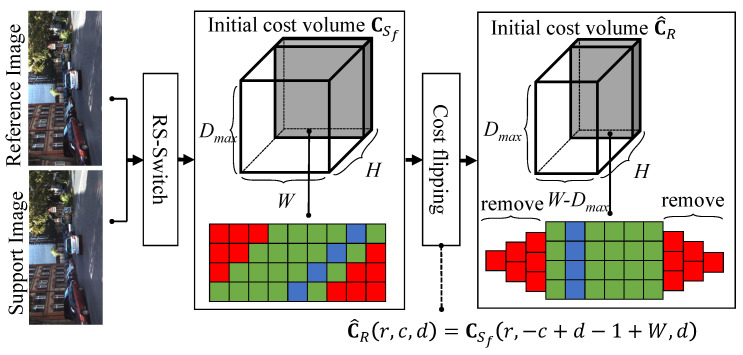
Illustration of initial cost volume consistentization, which consists of RS-Switch and cost flipping. The removed and active regions are marked in red and green, and the cost pillar is displayed in blue.

**Figure 4:**
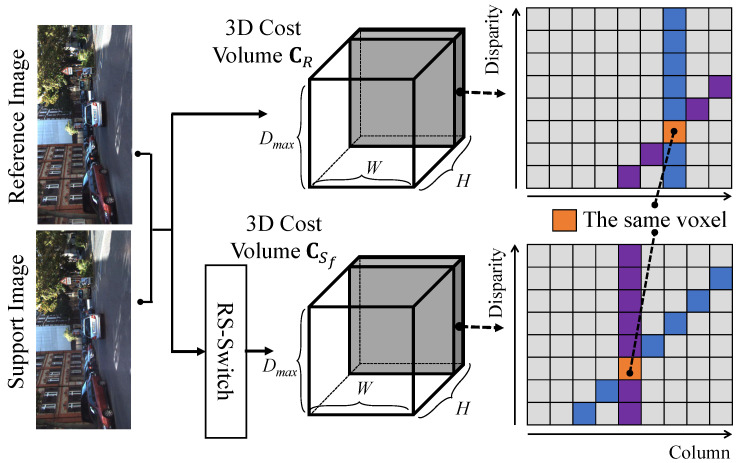
Illustration of cost volume inconsistency. A vertical cost pillar in $C_{R}$ is linked to a tilted cost pillar in $C_{S_{f}}$. The orange pixels in $C_{R}$ and $C_{S_{f}}$ are the same voxel.

Figure 3 illustrates the processes consisting of RS-Switch and cost flipping. The RS-Switch is performed before the generation of the initial cost volumes. The purpose of the RS-Switch is to exchange the reference and support images, such that the right and left images are selected as reference and support images, respectively. After exchanging the images, the exchanged images are horizontally flipped to ensure that the search directions in the epipolar lines are consistent in both matchings. However, after the processes of RS-Switch and feature extraction, the initial cost volumes from the left-to-right and right-to-left matchings (denoted as $C_{R}$ and $C_{S_{f}}$) are still inconsistent. For instance, in Figure 4, a vertical cost pillar, which is defined as the matching costs for a pixel in different disparities, in $C_{R}$ links to a tilted cost pillar in $C_{S_{f}}$, and vice versa. For instance, the orange pixels in $C_{R}$ and $C_{S_{f}}$ are the same pixels, but they are at different locations of the cost volumes. Therefore, a cost volume transformation is performed, and the initial cost volume $C_{S_{f}}$ is flipped according to the equation

(5) $$\begin{array}{c} {\hat{C}}_{R}\left(r,c,d\right)=C_{S_{f}}\left(r,-c+d-1+W,d\right), \end{array}$$

where ${\hat{C}}_{R}\left(r,c,d\right)$ represents the matching cost of pixel (r,c) in the reference image of the right-to-left matching, and this volume is called the dual cost volume of $C_{R}$. After the cost volume flipping, the border region of width $D_{max}$ (the red regions in Figure 3) are removed because of the incomplete information in the disparity axis.

### 3.3. Suppressed Regression

To take advantage of the left-to-right and right-to-left image matching, the cost volume $C_{R}$ and its dual volume ${\hat{C}}_{R}$ from Dual-GANet are merged and integrated. The merged cost volume $C_{M}$ is defined as follows:

(6) $$\begin{array}{c} C_{M}\left(r,c,d\right)=\left\{\begin{array}{cc} C_{R}\left(r,c,d\right), & \mathrm{if}\;c\in [0,D_{max}-1]\\ \frac{C_{R}\left(r,c,d\right)+{\hat{C}}_{R}\left(r,c,d\right)}{2}, & \mathrm{otherwise}. \end{array}\right. \end{array}$$

As $D_{max}$ pixels in the dual cost volume ${\hat{C}}_{R}$ have incomplete information in the disparity axis, the merged cost for those pixels directly assigns $C_{R}$ to $C_{M}$. Note that the cost volumes from left-to-right and right-to-left image matchings are regarded as the same after volume consistentization. Therefore, there are no parameters in the merge layer, and this layer is frozen during training for simplicity.

**Figure 5:**
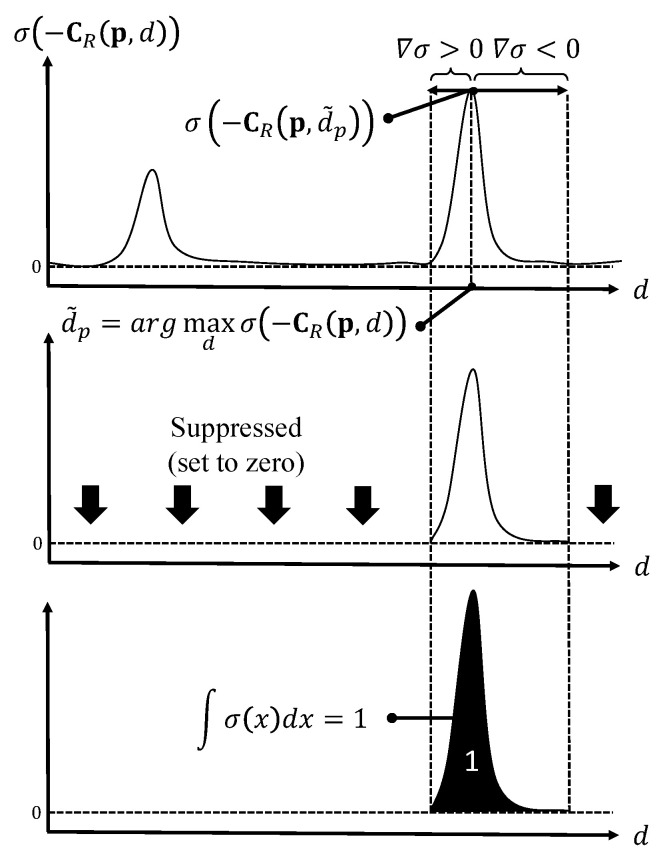
Illustration of the suppressed regression process. **Top**: determining the active regions; **middle**: suppressing the inactive regions; **bottom**: normalizing the active regions.

**Figure 6:**
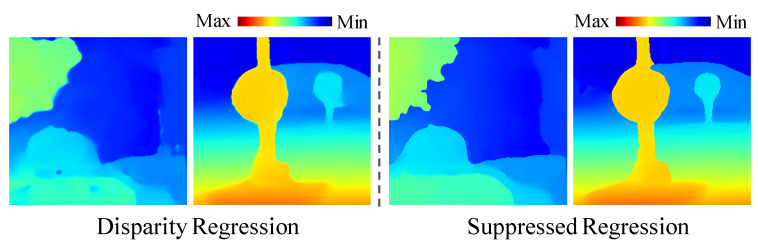
Comparison of the disparity regressions. The disparity maps are generated by the general regression (**left**) and suppressed regression (**right**). The disparity maps are visualized by colors ranging from blue (minimum disparity) to red (maximum disparity).

The disparity regression is generally performed for disparity estimation. However, the disparity regression relies on the generation of unimodal disparity probability distributions, and the regression of disparities may blur disparity edges. To alleviate the problems and to support multiple disparity candidates, an extension of the disparity regression called suppressed regression is proposed, which is similar to the idea of data suppression in [27]. As illustrated in Figure 5, the suppressed regression consists of four main steps to approximate the pixel disparities in subpixel accuracy. By following the disparity regression in [22], the predicted costs in the merged cost volume $C_{M}$ are converted into a probability volume by taking the negative of each value and normalizing the volume across the disparity dimension with the softmax operation σ(·). Then, the maximum of $\sigma (-C_{M}\left(p\right))$ is selected as the preliminary optimal disparity, denoted as ${\tilde{d}}_{p}$, for the pixel p. A neighboring region of ${\tilde{d}}_{p}$ is determined based on the gradient of the probability distribution, that is, ∇σ. The neighboring regions with ∇σ>0 and ∇σ<0 at the left-hand and right-hand sides of ${\tilde{d}}_{p}$, respectively, are selected as the active regions. The areas outside of the active regions are selected as inactive regions, and the disparities in the latter regions are suppressed to zero. The probability distribution is normalized in the next step, such that the integral of the active regions is equal to one. Then, the disparity regression is applied to the normalized active region. The neighborhood of the estimated disparity with near normal distribution is only used in the disparity regression. The possibility of acquiring accurate disparities can be improved owing to the removal of unrelated information prior to the disparity regression. A comparison between the general disparity regression and suppressed regression is shown in Figure 6. In this comparison, the feature edges of the disparity map from the suppressed regression are sharper than those from the general regression.

The idea of multiple disparity candidates comes from the top-*n* accuracy used in classification problems. To further measure the performance of the dual network and to fully utilize the information in multimodal disparity probability distributions, multiple disparity selection is provided. In this step, the active region is removed and then the process goes back to the first step for the selection of the next disparity candidate. The processes are terminated when all candidates are selected or when all the regions are removed.

### 3.4. Loss Function

Most deep learning models for stereo image matching use mean squared error (MSE) as the loss function and select softmax as the activation function in the output layer to generate sharp and unimodal disparity probability distribution. However, the use of MSE with sharp and unimodal probability distribution may lead to a slow convergence in model training. During back-propagation, most of the neurons in the output layer receive small gradients because of the unimodal probability distribution. The small gradients will slow down the updating of parameters. Following the design in classification, we alleviate this problem by using two-hot encoding with cross entropy as the loss function, and retaining the softmax activation function in the output layer. Due to the encoding and loss function, the small gradients can be avoided in the output layer and the parameters can be updated efficiently. In two-hot encoding, a disparity ground truth is encoded by two numerical values with weights. These two numerical values are the integers close to the disparity ground truth. The two-hot encoding is performed using the equation

(7) $$\begin{array}{c} y\left(d\right)=\left\{\begin{array}{cc} 1-d^{*}+\left\lfloor d^{*}\right\rfloor , & \mathrm{if}\;d=\lfloor d^{*}\rfloor \\ d-\lfloor d^{*}\rfloor , & \mathrm{if}\;d=\lfloor d^{*}\rfloor +1\\ 0, & \mathrm{otherwise} \end{array}\right.,\\ d\in \left[0,D_{max}\right],\;d^{*}\in \left[0,D_{max}\right), \end{array}$$

where y(d) is the encoded disparity probability distribution, and $d^{*}$ is a real value denoting the disparity ground truth.

The cross entropy is used as the loss function to measure the similarity between the ground truth and predicted disparities. This is defined as

(8) $$\begin{array}{c} L\left(y,\hat{y}\right)=\sum_{d=0}^{D_{max}}-\hat{y}\left(d\right)\ln y\left(d\right) \end{array}$$

where *y* denotes the probability distribution of the predicted disparity, which is the output of the softmax function σ(·), and $\hat{y}$ is the disparity ground truth encoded by two-hot encoding. Note that the selection of multiple candidates is performed in the evaluation phase. The encoding in Equation (7) and the loss function in Equation (8) uses a single disparity candidate in training.

## 4. Results and Discussion

The Dual-GANet is evaluated using the SceneFlow [12] and KITTI2015 [29] datasets. The model is trained using Nvidia RTX 2080Ti. Adam with the parameters $\beta_{1}$= 0.9 and $\beta_{2}$ = 0.999 is selected as the optimizer. The learning rate is set to 0.001 and 0.0001 for the SceneFlow and KITTI2015 datasets, respectively, and the batch size is set to 3 because of the limited GPU memory. The model is pre-trained and refined by using SceneFlow and KITTI2015, respectively. The total training time is 36 h, and the average inference time is 2.51 s for 256 × 1024 images, which are slower than that of the backbone GANet because of the dual network and left–right consistency learning scheme. The metrics used in the model evaluation include average endpoint error (average EPE), average error rate (average ER), and average confidence level (average CL). The EPE is defined as the absolute distance between an estimated disparity $\hat{d}$ and its corresponding ground truth *d*, that is, $EPE=\frac{1}{n}\sum_{i=1}^{n}\left|\hat{d}-d\right|$, where *n* represents the number of pixels. The error rate is defined with a threshold *k*, denoted by ER >k px, to represent the percentage of pixels with an EPE larger than *k* pixels. The confidence level is defined as the distance between the predicted disparities in the cost volume $C_{R}\left(p,{\tilde{d}}_{p}\right)$ and its dual cost volume ${\hat{C}}_{R}\left(p,{\tilde{d}}_{p}\right)$, which is used to measure the consistency of the left-to-right and right-to-left image matchings. In addition, the proposed model allows the selection of multiple disparity candidates, and the evaluation metric is applied to the best candidate.

### 4.1. Evaluation Using the Scene Flow Dataset

The input tensor is set to 192 (maximal disparity) × 512 (height) × 960 (width). Considering the limited GPU memory, the proposed model uses a downsized GANet, denoted as GANet_small, as the backbone. In GANet_small, the downsampling factor in the bottleneck layers of SGA is set to 3 instead of 4 in the GANet. Specifically, the tensors of the bottleneck layers in SGA are $\left(\frac{D_{max}}{4},\frac{W}{4},\frac{H}{4}\right)$ and $\left(\frac{D_{max}}{8},\frac{W}{8},\frac{H}{8}\right)$ instead of the original tensors $\left(\frac{D_{max}}{3},\frac{W}{3},\frac{H}{3}\right)$ and $\left(\frac{D_{max}}{6},\frac{W}{6},\frac{H}{6}\right)$, respectively. In addition, the number of layers in the feature extraction is reduced, and one-fourth of the original layers is removed.

**Figure 7:**
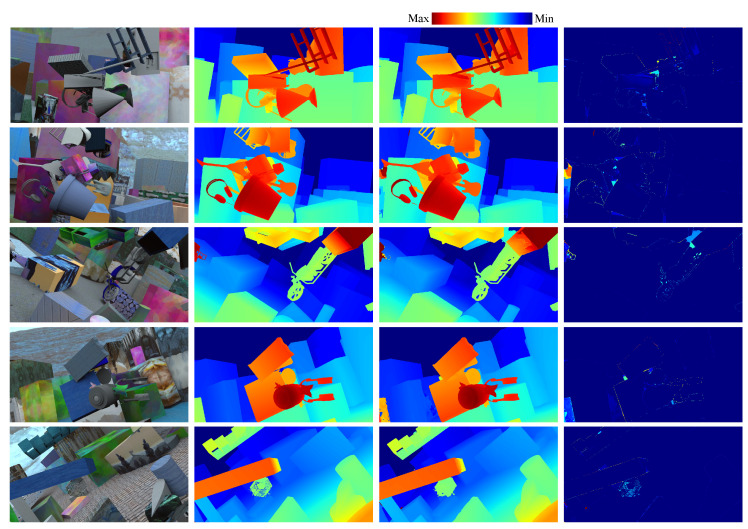
Disparity estimation using the SceneFlow dataset. First–fourth columns: input reference images; disparity ground truths; Dual-GANet results; and disparity error maps.

The disparity estimation results using Dual-GANet are shown in Figure 7. The evaluations of the six-direction 2D aggregation, suppressed regression, and multi-candidate selection are provided in Table 1. The results indicate that the average EPE and average ER in Model #3 are improved by 10.0% and 10.8%, respectively, compared with that of Model #2. This shows that the enlargement of the receptive field in the SGA layer can improve the estimation of disparities with relatively more semantic information. In Model #4, the average ER is improved from 10.31% to 7.37% compared with the result in Model #3, thereby demonstrating that the suppressed regression can reduce the error rate in the disparity estimation. In addition, in Model #6, the average ER is further improved from 7.37% to 6.65% with the aid of Dual-GANet. This shows that the flipped training for the learning of invisible-to-visible pixel matching and dual network scheme can reduce the error rate and mismatching. Furthermore, with the aid of multi-candidate selection, the average EPE and average ER in Model #7 can decrease to 0.418 px and 5.81%, respectively.

### 4.2. Evaluation Using the KITTI2015 Dataset

**Figure 8:**
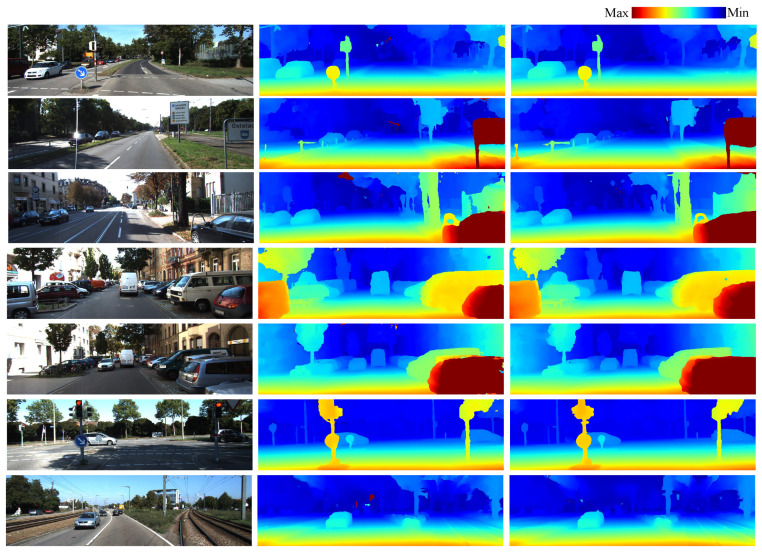
Disparity estimation using KITTI2015. **Left**: input reference image; **middle**: disparity results generated by GANet; and **right**: disparity results generated by Dual-GANet.

In the evaluation of the KITTI2015 dataset, the input tensor is set to 192 × 256 × 1024, and the six-direction cost aggregation and suppressed regression are used in both the GANet_small and Dual-GANet. The comparisons between the disparities estimated by GANet and Dual-GANet are shown in Figure 8. As shown in the visual comparisons, several mismatches occurred in the homogeneous sky regions in GANet_small. Most of these mismatches can be fixed using the Dual-GANet with left–right consistency learning. To further evaluate the proposed model, the related and recent methods and models, including SGM [2], GCNet [22], PSMNet [30], GANet [13], and CSPN [24], are compared. The comparison results are shown in Table 2. After comparing the Dual-GANet (with dual scheme) with GANet_small (without dual scheme), the results indicate that the average ER is improved from 2.32% to 1.76%. In addition, the Dual-GANet using a small version of the GANet as backbone is slightly better in terms of average ER than the original GANet. Furthermore, the Dual-GANet with two-candidate selection can reduce the average ER to 0.95%, which is better than the results of related models. Note that the dual scheme can be applied to other advanced models. The disparity estimation can be further improved when the full-size GANet or CSPN is used as the backbone.

### 4.3. Evaluation of Flipped Training

**Figure 9:**
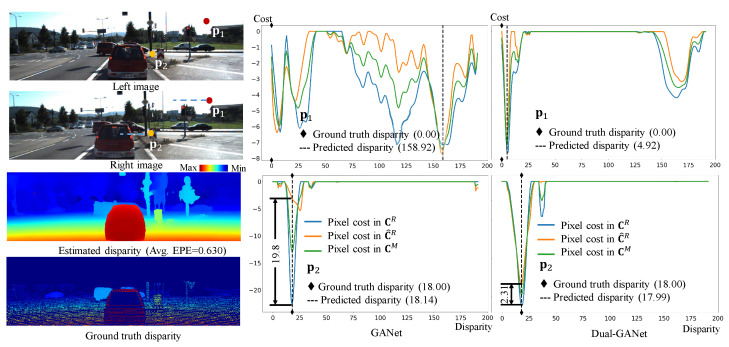
Comparison of the disparity probability distributions of pixels in the cost volumes of the GANet (**left**) and Dual-GANet (**right**).

To evaluate the dual scheme and flipped training, a comparison of the cost volumes generated by the GANet (without dual scheme) and Dual-GANet (with dual scheme) was conducted. The results are shown in Figure 9. The two points in the stereo image, denoted by $p_{1}$ and $p_{2}$, located in a homogeneous sky region and an occlusion region, respectively, were selected for evaluation. Their disparity search ranges along the epipolar lines are displayed as blue dots in the images. These two kinds of regions often cause mismatches because of poor context information and occlusion. Note that the disparity ground truth of $p_{1}$ is manually set to 0 because there is no ground truth available in sky regions. In Figure 9, the disparity probability distributions in $C_{R}$, ${\hat{C}}_{R}$, and $C_{M}$ are visualized by blue, orange, and green colors, respectively. For fair comparison, in the GANet, the cost volumes $C_{R}$ and ${\hat{C}}_{R}$ are generated separately. Then, the cost volume $C_{M}$ is obtained by merging $C_{R}$ and ${\hat{C}}_{R}$. In Dual-GANet, the cost volumes $C_{R}$, ${\hat{C}}_{R}$, and $C_{M}$ are generated using the workflow shown in Figure 1. The pixel disparities are obtained using the merged cost volume.

In the case of point $p_{1}$, the disparity probability distributions in $C_{R}$, ${\hat{C}}_{R}$, and $C_{M}$ of the GANet are inconsistent because of the poor context information in the homogeneous region, which results in unstable disparity estimation. The predicted disparity is 158.92, which is far from the ground truth (0.0). In contrast, with the aid of dual network and flipped training, the disparity probability distributions generated by the Dual-GANet are near consistent, and the estimated disparity (4.92) is close to the ground truth. In the case of point $p_{2}$, the disparity probability distributions in $C_{R}$, ${\hat{C}}_{R}$, and $C_{M}$ of the GANet and Dual-GANet are consistent. However, the confidence level (CL) in the Dual-GANet is 2.31, which is better than that in the GANet (CL = 9.8). This result demonstrates that the dual network and flipped training are able to stabilize the disparity estimation.

## 5. Conclusions and Future Works

A Dual-GANet for stereo image dense matching is proposed. By integrating the left–right consistent matching and utilizing the invisible-to-visible pixel learning in network design and training, the possibility of pixel mismatches can be reduced. In addition, the suppressed regression and multi-candidate selection can refine the disparity estimation and improve the probability of selecting the correct disparity. In the implementation, a downsized version of the GANet is used as the backbone with modifications on the loss function and aggregation. The uses of cross entropy with two-hot encoding and the six-direction aggregation with 3D receptive field in the LGA layers can reduce the matching error rate. The experiment results with the SceneFlow and KITTI2015 datasets demonstrate the feasibility and practicality of the proposed method. The compared models, including SGM, GCNet, PSMNet, GANet, and CSPN can reach satisfactory results on disparity estimation (average ER < 1.74%–6.38% for KITTI2005). With the proposed left–right consistent scheme, the average ER can be improved from 2.32% to 1.76%, compared with the backbone model. The limitation of the proposed and compared models is that deep learning models are designed and trained for close-range stereo images. In the near future, we are interested in extending the Dual-GANet to multi-image matching. In addition, unsupervised learning techniques based on spatial transformation and view synthesis are considered for integration in network training.

## Figures and Tables

**Table 1:** Evaluation and comparison using the SceneFlow dataset. The number of aggregation directions is denoted as # of A.D.

No.	Model	# of A.D.	Disparity Regression	Suppressed Regression	2-Candidate	Average EPE (px)	Average ER > 1px
1	GANet	4	✓			0.780	8.7%
2	GANet_small (backbone)	4	✓			0.995	11.56%
3	GANet_small	6	✓			0.895	10.31%
4	GANet_small	6		✓		0.865	7.37%
5	GANet_small	6		✓	✓	0.440	6.56%
6	Dual-GANet	6		✓		0.862	6.65%
7	Dual-GANet	6		✓	✓	0.418	5.81%

**Table 2:** Comparisons of the related methods and the proposed models using the KITTI2015 dataset.

Model	Average EPE	Average ER > 3 px
SGM [2]	–	6.38%
GCNet [22]	–	6.16%
PSMNet [30]	–	2.32%
GANet [13]	–	1.81%
CSPN [24]	–	1.74%
GANet_small	0.790	2.32%
Dual-GANet (single candidate)	0.712	1.76%
Dual-GANet (two candidates)	0.589	0.95%

## Data Availability

Not applicable.
